# Communicating diagnoses to individuals with a first episode psychosis: A qualitative study of individuals perspectives

**DOI:** 10.3389/fpsyt.2023.1098224

**Published:** 2023-02-16

**Authors:** Marina Elisabeth Huurman, Gerdina Hendrika Maria Pijnenborg, Bouwina Esther Sportel, Gerard David van Rijsbergen, Ilanit Hasson-Ohayon, Nynke Boonstra

**Affiliations:** ^1^Friesland Mental Health Care Services, Leeuwarden, Netherlands; ^2^Department of Clinical Psychology and Experimental Psychopathology, University of Groningen, Groningen, Netherlands; ^3^Department of Psychotic Disorders, GGZ Drenthe Mental Health Institute, Assen, Netherlands; ^4^Department of Psychology, Bar-Ilan University, Ramat Gan, Israel; ^5^Department of Healthcare and Welfare, NHL Stenden University of Applied Sciences, Leeuwarden, Netherlands; ^6^KieN VIP Mental Health Care Services, Leeuwarden, Netherlands; ^7^Department of Psychiatry, UMC Utrecht Brain Center, University Medical Center Utrecht, Utrecht, Netherlands

**Keywords:** psychosis, communicating, stigma, needs, individual’s perception

## Abstract

**Background:**

Receiving the label of a psychotic disorder influences self-perception and may result in negative outcomes such as self-stigma and decreased self-esteem. The way the diagnosis is communicated to individuals may affect these outcomes.

**Aims:**

This study aims to explore the experiences and needs of individuals after a first episode of psychosis with regard to the way in which information about diagnosis, treatment options and prognosis is communicated with them.

**Design and methods:**

A descriptive interpretative phenomenological approach was used. Fifteen individuals who experienced a first episode of psychosis participated in individual semi-structured open-ended interviews on their experiences and needs regarding the process of providing information about diagnosis, treatment options and prognosis. Inductive thematic analysis was used to analyze the interviews.

**Results:**

Four recurring themes where identified (1) *timing* (when); (2) *content* (what); and (3) *the way information is provided* (how). Individuals also reported that the provided information could elicit an emotional reaction, for which they would require specific attention, therefore the fourth theme is (4) *reactions and feelings*.

**Conclusion:**

This study provides new insights into the experiences and specific information needed by individuals with a first episode of psychosis. Results suggest that individuals have different needs regarding the type of (what), how and when to receive information about diagnosis and treatment options. This requires a tailor-made process of communicating diagnosis. A guideline on when, how and what to inform, as well as providing personalized written information regarding the diagnosis and treatment options, is recommended.

## Introduction

Receiving a classification of a psychotic disorder has several implications, potentially resulting in self-stigma and decreased self-esteem (1). The way a diagnosis is communicated to individuals is central to mental health practice and has the potential to influence individuals outcomes, including treatment engagement, willingness to follow treatment recommendations, satisfaction with treatment, symptom severity, and referral to other services (2). Although there is limited literature on how and when to inform individuals after a first episode of psychosis (FEP) about their diagnosis, treatment options and prognosis; the Dutch guideline on early psychosis recommends working with a personalized diagnosis to guide treatment, while using a DSM classification for insurance purposes [e.g., (3)]. A personalized diagnosis involves a description of the formal diagnosis together with a description of personal vulnerability, triggering, maintaining and protective factors. It also includes considering how symptoms affect functioning and wellness and personal treatment options. According to the guideline, diagnosis of psychosis includes three components: an individual, a dimensional and a categorical profile (3). The individual profile maps out which factors are (or have been) influencing the onset, provocation and maintenance of the psychotic episode and what the relevant protective and vulnerable characteristics were. A dimensional profile describes the extent to which certain symptoms are present. Finally, a categorical description serves to determine whether the symptoms fall within the definition of a particular psychiatric disorder, for example a DSM 5 classification. The individual, as well as the dimensional and the categorical profile are all integrated in a personalized diagnosis that is communicated to the general practitioner through a letter. The guideline recommends that this letter should be composed in consultation with the patient.

In psychotic disorders, communication regarding diagnosis is complicated by several factors. First of all, at early stages, prognosis may be uncertain and course of illness and outcomes may vary substantially (4–6). *Secondly s*chizophrenia and other psychosis-based diagnoses have no objective evidence (e.g., blood test, imaging) and depends on the clinical judgment of behavioral criteria, which, in addition, can be influenced by cultural biases (7, 8). Thirdly, diagnostic labels such as “schizophrenia” are heavily stigmatized (1). Considering all these factors that can affect the process of diagnosis communication, it is important to explore the subjective experience of individuals regarding the way they learned about their diagnosis and the implications of this process.

The way information on diagnosis, treatment and prognosis is communicated, can impact a person’s recall and understanding of the diagnosis, the treatment options and prognosis, and how they accept the diagnosis and treatment plan, which then ultimately can affect the process of recovery and long-term outcome (9). When information about their illness is not communicated adequately individuals may feel confused and perplexed (10). As a consequence, communicating diagnostic information relating to psychotic disorders in particular is a complex task for mental health workers (9, 10). Especially in the early phase of illness, when individuals receive information on their disorder for the first time, providing clear, comprehensive and concise information in a respectful way seems crucial.

A recent study on clinicians working in Early Intervention Services (EIS) highlights the challenges posed in discussing the diagnosis of FEP (10). Most clinicians identified a number of barriers, such as diagnostic uncertainty, stigma and the variations in outcome. Therefore, the term “psychosis” or FEP was preferred (10). Because cognitive deficits, impaired metacognition and insight can interfere with the communication and interpretation of information, the need for information on the diagnosis of FEP for those experiencing psychotic symptoms is completely different from other serious disorders (2, 10, 11). In line with the preferred term psychosis or FEP, it has been debated whether the term schizophrenia should be replaced with psychosis spectrum syndrome (12–14). It seems that health care providers are avoiding the term schizophrenia in their communication, but not in the individuals’ file, and that a diagnostic label, such as schizophrenia, may be discovered by individuals accidentally in their medical file.

There is a paucity in literature of information on the process and experiences of communicating about diagnoses that takes place in psychiatry, and, consequently, on recommendations regarding how to communicate which information and when (15). Given the plethora of research in other medical disciplines, such as oncology, pediatrics, emergency settings and neurology (16–18) it is surprising that in psychiatry, research on communicating information about a serious illness is so scarce. However, there are some studies about delivering difficult news in psychiatry showing that individuals diagnosed with mental disorders and their relatives wish, in line with their rights, to be fully informed on their diagnosis and it implications (19, 20). However, limited studies on this topic showed that individuals and their relatives have expressed dissatisfaction with the way diagnostic and related information has been shared with them in psychiatry (21, 22) and showed the need for such protocols (23). Thus, it is important to further learn on how and when to inform individuals with a first episode about their diagnosis and related information on prognosis in order to design protocols for communication about diagnoses. The current study aims to gain insight into the lived experiences and needs of individuals after FEP, regarding the process of providing information about diagnosis, treatment options and prognosis, using an interpretative phenomenological approach.

## Materials and methods

### Study design

In this qualitative study, a descriptive interpretative phenomenological approach was used (24), conducting in-depth interviews to gain insight into the lived experiences, experienced of individuals, after FEP, aged above 18 years of age. In this study we aimed to understand the subjective experiences and needs regarding the process of providing information about diagnosis, treatment options and prognosis after a first episode psychosis. Due to the exploratory nature of the study, a qualitative study is best suited to help us provide unique insights and uncover unexpected findings (25).

Accordingly we conducted in depth interviews 6–12 months after the psychotic episode. This range was chosen since communicating diagnosis is part of a diagnostic process and not based on one single conversation event. In addition, at the first contact (intake), most patients are having psychotic symptoms and learning to understand symptoms in relation to its context is part of the diagnostic process which takes a few months. We were looking for the subjective experiences of patients and not for the objective situation nor for the perspective of the clinician.

### Participant selection

The study population consisted of individuals after FEP, treated by the early intervention service (EIS) of Friesland Mental Health Care Services or the EIS of Drenthe Mental Health Care Services in the Netherlands. An EIS provides outreaching care for individuals with an early psychosis (26). A convenience sample, based on availability and willingness to participate, was assembled. All individuals from 18 to 65 years, who entered treatment for a first episode psychosis were asked by their psychiatric nurse to participate in this study between 6 and 12 months after the intake. 6–12 months was chosen because at the first contact (intake), most patients are having psychotic symptoms as this is the reason for their referral. Learning to understand symptoms in relation to its context is part of the diagnostic process and takes a few weeks or months.

The nurses asked the participants if they wanted to share their experiences about the way they were talked to about their psychiatric diagnosis and what could have been improved. Individuals with florid psychotic symptoms, according to their clinicians, were excluded from participation. After individuals had indicated that they wanted to participate in the study, they were contacted by telephone by the researcher to receive further explanation. After consent to participate, an appointment was made for an interview. Participants were interviewed once because with this in depth interview we aimed to question the participants about the whole diagnostic period. Since the focus of this study is on the first phase after intake, there was no follow up assessment. Participants were, however, asked to review the interview and add additional insights.

### Semi-structured interview

The experience of communicating the diagnosis was assessed by applying a semi-structured open-ended interview guide, developed by Amsalem et al. (21). It focused on individuals experiences of the way the diagnosis was communicated to them by their treating clinicians. The interview included twelve core questions and nineteen sub questions, that were used flexibly. Examples of the questions are: (1) “Can you tell me a bit about who you are?” (2) “Why do you think you are being treated here?” (3) “What have you been told about the reason why you are being treated?” (4) “How did you come to know this?” (5) “Was the information on…. sufficient, too little or too much?” (6) “What did you think of the timing of the conversation about the diagnosis?” The interviewer used an active listening style to establish rapport.

### Data collection

The interviews were conducted by two experienced mental health care psychologists. We used the COnsolidated criteria for REporting Qualitative research (COREQ) checklist to make sure all of the items from the checklist were included in this qualitative research (27). All participants gave written informed consent prior to participation. The interviews were conducted online or at their local clinic, depending on the respondent’s preference. The interviews were audio-recorded and transcribed verbatim. The duration of the interviews was between 30 and 90 min. The iterative process of sampling, data collection and analysis was continued until data saturation was reached. The term “data saturation” was defined as the point in data collection and analysis when new incoming data produces little or no new information to address the research question (28). After fifteen interviews, when three further interviews had been conducted which produced no new themes, we designated this as the point of data saturation.

### Procedure and ethical considerations

For this study, which was part of a larger study, the research proposal was submitted to the Medical Ethics Committee of the University Medical Centre (MEC) for approval. The committee granted the approval, registered under no. NL64406.042.18. The recordings of the interviews are retained according to the international safety regulations for the storage of data at the University of Groningen and NHL Stenden University of Applied Sciences and are accessible only to the researchers.

### Data analysis

Authors MH and NB analyzed the data and coded them independently. Transcripts were analyzed thematically, using the widely used approach of Thematic Analysis (TA) by Braun and Clark (29) to organize, encode and identify patterns within qualitative data. Braun and Clarke (28) distinguish between a top-down or theoretical thematic analysis, that is driven by the specific research question and the analyst’s focus, and a bottom-up or inductive one that is more driven by the data itself. Our analysis was driven by the research question and thus was more top-down than bottom-up. The theme that was established *a priori*, included individuals’ experiences and needs regarding communicating diagnosis (including treatment options and prognosis) (10, 30).

Step 1 involved listening to the audio tape whilst reading the verbatim interviews (data familiarization). Data relevant for each code were collected in step 2 (extracting significant statements). Then in step 3, we compared the codes with the formulated meaning (based on the quotes) to achieve consistency (compare and discussion). During step 4, we categorized the meanings into codes that reflect a vision to form a code (27 codes were obtained from 141 meanings which were incorporated into 4 themes). In step 5, we generated clear names for each theme. Finally, in step 6, we discussed whether the selected quotes, codes and themes all relate back to the research question. The researchers compared their results and discussed differences and agreed on a common analysis. If there would have been a disagreement a third researcher (MP) would have added to the discussion but this was not the case.

Table 2 shows the themes and codes that were identified, along with the examples of quotes illustrating them.

**TABLE 1:** Participant demographics, medications, self-reported diagnoses and DSM 5 classification (*N* = 15).

Participant	Biological sex	Age	Residential status	Medications	Self-reported diagnoses	DSM 5 classification	Treatment
1	M	33	Living alone	Olanzapine	“I can’t describe it, I don’t have a psychiatric disorder”	Unspecified psychotic disorder or other psychotic disorder (298.8)	Crisis assessment and treatment team admission to a psychiatric hospital EIS
2	V	38	With partner and children	Olanzapine	“There was just too much going on in my head. Really too many problems”	Short-term psychotic disorder: with obvious stressor(s) (298.8)	EIS
3	M	21	With parents	Olanzapine	“I have been in a psychosis, my whole world was different. I had a lot of thoughts that weren’t right”	Short-term psychotic disorder: with obvious stressor(s) (298.8)	Admission to a psychiatric hospital EIS
4	V	30	With partner	Olanzapine	“I have been in a psychosis”	Unspecified psychotic disorder or other psychotic disorder (298.8)	Crisis assessment and treatment team EIS
5	M	25	Living alone	Olanzapine	“It’s just there, learning how to live with a psychotic vulnerability”	Short-term psychotic disorder: with obvious stressor(s) (298.8) Autism spectrum disorder (299.00)	Crisis assessment and treatment team Admission to a psychiatric hospital EIS
6	M	40	With partner and children	Olanzapine	“Psychosis”	Short-term psychotic disorder: with obvious stressor(s) (298.8)	Crisis assessment and treatment team EIS
7	M	40	With partner and children	Olanzapine	“Psychosis”	Other specified psychotic spectrum or other psychotic disorder (298.8)	Crisis assessment and treatment team EIS
8	M	30	With partner and children	No medication	“I don’t think I have a disorder”	Short-term psychotic disorder: with obvious stressor(s) (298.8)	EIS
9	V	32	Living alone	Quetiapine	“Social anxiety and recovering from a psychosis”	Short-term psychotic disorder: with obvious stressor(s) (298.8) Major depressive disorder: single episode, moderate (296.22)	EIS
10	M	29	Living alone	Olanzapine	“Feeling of being chased, that a lot of people wanted something from me”	Unspecified psychotic disorder or other psychotic disorder (298.8)	Admission to a psychiatric hospital EIS
11	M	21	With parents	Olanzapine	“Drug induced psychosis”	Psychotic disorder due to other hallucinogens (292.2)	EIS
12	M	32	With parents	Olanzapine	“Psychological delusions, kind of”	Unspecified psychotic disorder or other psychotic disorder (298.8)	EIS
13	M	25	Living alone	No medication	“Mild mental disorder’	Unspecified psychotic disorder or other psychotic disorder (298.8)	EIS
14	V	32	Living alone	Risperidone	“Comes from childhood, hear voices in the past”	Unspecified psychotic disorder or other psychotic disorder (298.8)	Crisis assessment and treatment team admission to a psychiatric hospital EIS
15	M	35	Living alone	Methylphenidate	“ADHD and psychosis”	Schizophreniform disorder (295.40) Attention deficit hyperactivity disorder (314.01)	EIS

EIS, early intervention service (outreaching treatment).

**TABLE 2:** Experiences of the communication of diagnostic information in patients after FEP: examples of the phenomenological process using thematic analysis [Braun and Clark (29)].

Thema	Codes	Quotes
1. Timing	Overwhelming during psychosis When the patient is ready As soon as possible	“The timing depends on the person and it is up to the clinician to judge what the right timing is” (participant 1) “I thought the psychiatric interview was some kind of joke or something. I didn’t take it seriously because I was in my own world” (participant 3). “I’m not interested in the diagnosis. For me it’s more that I just want to get my life back to normal. And I think that’s more important than the diagnosis” (participant 10).
2. Content of received and desired information	Didn’t remember a lot of the conversation No structural approach Never been informed Too little information was given during the psychiatric interview on the purpose and outcome The letter to the general practitioner came as a surprise	“I asked for it after 3 weeks. I want to know what’s going on with me, I like clarity.” (participant 5) “I don’t really remember, but I have to say that the diagnosis, like “you have this”, had never really come out very strongly. I don’t think it’s been until the last few weeks, that we’ve talked about it.” (participant 8) “They never told me that a letter would be sent to the general practitioner. The content of the letter was confrontational”. (participant 5)
3. Way of providing information	Didn’t want a lot of information Would like to have the choice when the information is given Need clarity and explanation Preference for more written information Family support A connection with the clinicians who performed the psychiatric interview Notified of the letter to the general practitioner A cozy environment Pay more attention to strengths/qualities Listening The caregiver’s tone of voice Ask more about the cause Need for practical information Need for hope	“I think it’s nice to have something on paper. That there may actually be a concrete plan so you have something to hold on to”. (participant 2) “I was very sensitive to stimuli. I remember wearing sunglasses for the lights in the living room for days at night. So sitting under fluorescent lights during the psychiatric interview was not very pleasant, I would prefer a cozy environment” (participant 3) “At that time I was very sensitive to all kinds of impressions, to sound, light and emotions and I had the feeling that I really felt the energy of people. I didn’t have a good “click” with my clinicians who performed the psychiatric interview and that was an unpleasant experience”. (participant 1) “I missed a professional judgment. I had a lot of conversations at the time, and people also came by, all good conversations but I missed the purpose of the conversations” (participant 7) “The information was sketched out on paper. Maybe this could be more clearly designed so that the information sticks better”. (participant 11) “I didn’t need much information about how your brain works but how I could deal with my disorder”. (participant 13) “Keep in mind that different people have different needs. Maybe one person doesn’t think the diagnosis is that important, well then you don’t have to talk about it for long. But if someone else does think it’s important than it needs attention” (participant 9)
4. Reaction on received information	Clarification gives rest, relieve- and understanding Clarification leads to irritation, stigma and insecurity. Receiving information is confronting and leads to distrust It is intense, emotional and overwhelming. Insight gives hope	“It was emotional and confrontational to talk about the diagnosis”. (participant 4) “What I remember is that I wanted them to stop asking questions and help me to get some peace of mind”. (participant 5) “All of a sudden, I was so irritated and I just needed to know what was going on with me. Fill me in, because in the end, the story is about me.” (participant 7) “She tried to avoid the word schizophrenia and that was actually stigmatizing” (participant 9)

### Trustworthiness and authenticity

To ensure the criteria of credibility, transferability, dependability and confirmability, a series of actions was carried out (29). We used a semi-structured interview guideline to ascertain we asked the same questions to all participants; we included a heterogeneous group with respondents as representative as possible on age, gender and residential status of the whole group of individuals with FEP; we recorded, transcribed verbatim and returned a summary of answers to obtain confirmation of the participants that the transcribed data was accurate; the analysis was carried out independently by two researchers and the total research team participated in the process of validation of the results; the characteristics of the participants were reported in detail; the findings where accompanied by quotes from the participants.

In order to achieve *authenticity*, every effort was made to fairly represent the experiences of the participants. A member check technique was applied to validate the findings. During the feedback sessions, individuals reported that the researchers understood their experiences correctly, indicating the information was interpreted correctly.

## Results

Fifteen individuals (11 males, 4 females) between 18 and 40 years old (*M* = 30.5, SD = 6.04) took part in the current study. Interviews were carried out between December 2020 and May 2021, resulting in approximately 11 h of audiotaped interviews (range: 23–72 min), transcribed in 162 pages. Table 1 presents participants’ demographics, residential status, medications, self-reported diagnoses, DSM classification according to the medical file and treatment setting(s).

During the data analysis, four themes were identified as relevant to the experiences and needs of individuals after FEP regarding the communication of diagnostic information relating to the psychotic episode: (1) *timing* (when); (2) *content (what);* and (3) the *way* information is provided (how).

Individuals also reported that the provided information could elicit an emotional reaction, for which they would require specific attention, therefore the fourth theme is (4) *reactions and feelings*. The themes are described in more detail below. Table 2 gives an example of the phenomenological process.

### Timing – The moment information is given

Individuals were asked to describe what they remembered from the conversation(s) about the diagnosis. Six individuals (40%), who had contact with crisis assessment and treatment teams or were admitted to a psychiatric hospital before they came under treatment at the early intervention service (EIS), said that they actually did not remember much of the conversations about the diagnosis, for all they knew, it might not even have taken place at all. These individuals said they were in the acute phase of psychosis at that time. They indicated that they did not need a lot of information, rather they wanted rest. When individuals came to the EIS for the initial psychiatric interview, individuals generally experienced less psychotic symptoms, they especially needed clear information about the purpose of the psychiatric interview, diagnosis, and related information on treatment options and prognosis.

*“I think they have told me before, but I was psychotic at that time. I just don’t have a clear memory of it.” (participant 14)*

Opinions on the timing of communication related to diagnosis, treatment options and prognosis were diverse. Ten individuals indicated that they would have preferred the information about the diagnosis at a time convenient for them, or as they said “*when I was ready for it myself”* (participant 5). This would have been after the most severe psychotic symptoms had disappeared. Three individuals (20%) indicated that they would have liked to have been informed about the diagnosis as soon as possible. These individuals were no longer in the acute phase at the time of admittance to the EIS. Only two individuals (13%) said that the timing of information about the psychiatric diagnosis did not matter to them. However, all individuals indicated that they would like to have had the choice whether and when they would have been informed about the diagnosis.

*“That will differ from person to person, but for me, the moment that I had calmed down a bit. I wouldn’t mention the classification in the beginning, then you see the world very differently and you are still very confused. For me, it would be 3 weeks later or so, then it dawned on me, that would have been the time for me to clarify things.” (participant 5)*

*“I would prefer the option that if you are ready, they can provide information. Because at the moment, you may be completely lost. Then you’ve lost touch with reality.” (participant 6)*

### Content – The content of the information given

The purpose of the psychiatric interview at an Early Intervention Service, namely to come to an individual, dimensional and categorical profile, was unclear to almost everyone, except for the individuals who were referred from a crisis assessment and treatment team to the EIS. In addition, most individuals mentioned that little information on DSM 5 classification and diagnosis was provided during the psychiatric interview. Individuals reported various ways of communicating the diagnosis, no structural approach was described. Only three (20%) individuals said they received information about DSM classification during the intake phase (first three to 6 weeks) at the EIS. Other individuals received information about the DSM 5 classification at a later phase during their treatment. Three individuals (20%) reported that they had never been informed about the categorical description until their participation in the current study. Two individuals (13%) had to ask about the categorical classification explicitly, because they had not been informed.

Twelve individuals (80%) were not informed about the content of the letter that was sent to the general practitioner after the psychiatric interview. Three individuals (20%) were informed about the content of the letter. This letter contained a description of the individual, as well as the dimensional and the categorical profile, based on the psychiatric interview. The content of this letter came as a surprise for these individuals. The content was often confrontational because the information had not been shared with them before. They felt they should have been prepared for the letter. In addition, schizophrenia was often described in the letter as a differential diagnosis while they were only informed about a first episode psychosis.

*“I don’t think they mentioned it during the intake.” (participant 2)*

*“I did not get any information during the first appointment. I was mainly asked to give a lot of information at the time and did not receive information. My partner was also asked how she looked at me. At that time, not much was shared with me. I was being questioned. I thought, why should I, there’s nothing wrong with me. I wasn’t in the mood for it at all.” (participant 7)*

“*It would have been nice to have been told, for example, that certain things are part of it (the disorder) and that these experiences are normal. You need the confirmation that you are not going crazy, I kind of missed that. I thought I was going insane.” (participant 5)*

### The way information is provided

About half of the individuals (*n* = 7, 46%) indicated that they would have liked more written information so they could reread it and did not have to retrieve it from memory, since they did not fully trust their memories. Individuals indicated that they were satisfied with the treatment, however, that the reason for treatment was not always clearly communicated to them. They all needed clear information but the needs in amount of information and when it should be given were all different.

*“What I needed at the time, was a piece of paper about what was wrong with me. That I just got a letter saying what’s wrong with me. I haven’t seen any of these.” (participant 3)*

*“I would have liked more treatment options and things on paper, I like to have an overview.” (participant 7)*

All individuals described that they would have liked additional information and support from their mental health care worker, like family support, practical support, more attention to causes and more attention to personal strengths and qualities. The connection with the clinicians who conducted the psychiatric interview was deemed an important factor, as well as the tone of voice used to provide the information. Two individuals (13%) would have preferred a more homely atmosphere with dimmed lighting, because they were so sensitive to stimuli. Three individuals (20%) described that they found the presence of family during the psychiatric interview very important.

### Reaction to the received information

The majority of individuals (*n* = 8, 53%) indicated that it was a very intense experience to be informed that they had a first episode psychosis and they often experienced negative associations with this term. However, most individuals (*n* = 13, 86%) also indicated that, despite the fact that the conversation about the diagnosis was confrontational, they liked to receive clarity because it provided rest, relief and understanding. Others said that this clarity resulted in irritation, stigma and insecurity. One of the individuals reported a lack of clarity as stigmatizing. Of the twelve individuals (80%) who had received a DSM 5 classification, nine of them agreed with the label. They reported that when they calmed down, over time, it was easier for them to accept the diagnosis. They became calmer after the medication started working and their sleep improved. The reaction of individuals loved ones played an important role in the acceptance of the diagnosis.

*“That was shocking, a schizophrenic disorder sounds more intense than a psychosis.” (participant 4)*

*“It was confronting but I like clarity the most.” (participant 5)*

*“Well, I think psychosis is a strong word. Then you think, I’m not mentally well, so uhm. yes. I thought that was quite intense. That I would be mentally ill, and that obviously doesn’t fit in the life I lead, with a busy job and a busy family.” (participant 7)*

Ten individuals (67%) reported that they were satisfied with the overall diagnostic and treatment process. During treatment, clinicians were said to only have paid attention to reactions on diagnostic information when individuals brought it up for discussion. Individuals reported that they experienced no initiative from clinicians to talk about emotions regarding the diagnostic information they had just received.

*“I found a bit of peace when a diagnosis was made. I could then let go of a kind of struggle and I no longer went looking for what could actually be going on.” (participant 5)*

*“It was confronting, but I needed the clarity. I just wanted to know where I stood. Even if that is confronting, I just needed to know the facts, because that means the most to me.” (participant 9)*

*“The diagnosis gave me peace of mind, I am not crazy, I am not the only one.” (participant 10)*

## Discussion

To the best of our knowledge, this is the first study to investigate the experiences and preferences of individuals in early intervention services, related to receiving the diagnosis of a FEP. Participants reported that timing (when), content (what) and the way information is given (how) was important for them. The specific needs in that respect varied from person to person. Individuals did, however, report that they experienced no initiative from clinicians to talk about their reaction to the diagnosis. Individuals were informed in different ways and at different times about their diagnosis, although some of them were informed not at all about their diagnosis and treatment options, or they could not remember what kind of information was provided. Participants also reported a lack of written information and none of the participants received a document regarding their diagnosis that was subsequently discussed with them by the professional.

Participants who received a psychotic spectrum diagnosis indicated that, after the start of the treatment, they first wanted some rest to clear their mind before receiving further information on their condition. Participants reported a different need during the acute phase and the phase in which acute symptoms reduced in intensity. Individuals in the acute phase, often treated by a crisis assessment and treatment team, could not remember much of the information they received about the diagnosis. At such times, they especially needed rest and were not able to process a lot of information. As established in Leonhardt et al. (31) metacognition and insight during the acute phase might have interfered communication.

Once the acute phase was in early remission and individuals were referred to the EIS, almost every participants (*n* = 13) had wanted to have received more information about the diagnosis, some of them preferably in writing. This corresponds with previous research, suggesting individuals with psychosis prefer an individual descriptive diagnosis, in writing, no matter how negative, to the alternative of struggling with uncertainty and unclarity (19, 32, 33).

Participants indicated that, at the time of receiving the information, they became demoralized by the information and therefore sometimes wanted to hold it off. Caregivers may consider motivational interventions, like motivational interviewing, in order to motivate their patients to talk about diagnosis and treatment options (33). Almost all participants who were treated by one of the EIS reported that receiving information about the diagnosis was important to them. Participants reported various ways in which the diagnosis was communicated, however, the tone, feeling a connection and non-verbal communication were deemed important. Our findings are in line with the findings of Milton and Mullan, who highlighted that transparent information sharing, conveying hopeful information, utilizing collaborative approaches and actively addressing stigma are vital to the diagnostic communication process (10, 34, 35).

We found that the main barrier for withholding the diagnosis schizophrenia is the stigma attached to the term “schizophrenia.” Participants in our study mentioned stigma as an important factor as well, although they also reported experiencing stigma from the professional who would withhold information. The second most important barrier reported by Farooq et al. (10, 35) was the fear that informing individuals about this diagnosis could be harmful to the individuals. This fear was felt by individuals and gave them the feeling it was serious. The third reported barrier for lack of guidance in mental health on how to communicate diagnosis in individuals with schizophrenia also applied in our sample: participants mentioned that there was no (structured) approach whatsoever, as a consequence whereof they often received information about their diagnosis coincidentally or even had to actively request it.

The current research shows that individuals actually need clarity, even if it is confrontational. They would like to have a choice when to be informed, because that preferred moment is different for everyone. The lack of metacognition (not knowing when you are ready) might interfere in this respect, as individuals might be able to indicate afterward that they would have liked to have known more, however, during the psychotic episode they might have been reluctant. In other words: it sometimes seems easier to assess when information would have been due in retrospect than when someone is in the process. This requires clinicians to repeat their information offers and keep tuning in on information needs, which we realize is a challenge for clinical practice. This research shows the importance of offering clarity through shared decision-making. Shared decision-making requires that sharing information with the individuals and their relatives about the diagnosis and outcome is part of treatment planning, based on open and honest communication about the diagnosis and outcome and about the uncertainties in the present state of knowledge (35). Engaging in shared decision-making (SDM) has been recently recognized as an integral strategy for helping individuals with serious mental illnesses choose the best treatment for them, increase adherence to their treatment decision and improve mental health outcomes (36, 37). The study of Hamman et al. (35) showed that individuals with schizophrenia have a slightly stronger preference for SDM than primary care individuals. Among those with schizophrenia, younger individuals and those with more negative views on medication want more participation. This might also apply to individuals with FEP. Despite the proliferating development of SDM interventions, SDM implementation for individuals with serious mental illness has not been successfully implemented everywhere. This has to do with sufficient workforce, leadership and financial circumstance in a department (38). Zisman-Illani et al. (39) offer a new conceptualization, shared risk-taking, as a unique way to view SDM with individuals with serious mental illness. They argue that risks are an inevitable part of the SDM process and are not only related to the decision at hand. Furthermore, they argue that joint reflection by the clinician and patient should be used to address SDM-related risks (40). Also (online/mobile) health interventions are developed to improve SDM (41).

Most participants were eventually, later on in the treatment, informed about treatment options and prognosis regarding the psychosis. The fact that psychosis is not a diagnostic category in classification systems such as DSM 5 poses the problem that individuals accidentally discovered another diagnostic label, such as schizophrenia or bipolar disorder with psychotic features, *via* the letter that is sent to the general practitioner. Our study shows that only one of the individuals were informed about what was communicated to their general practitioner. This may create confusion, as the professional diagnostical terminology may be confrontational if read without further detailed explanation. SDM regarding the sharing of the information also seems important and may actually contribute to reducing stigma. It is unclear why care providers do not discuss the contents of the letter with their patient before sending it to the general practitioner. To investigate this, interviews with caregivers should be conducted.

Only few protocols have been developed to facilitate the challenging process of information disclosure, one of the best known and most elaborate is the SPIKES protocol (42). The principles in this protocol align with the principles that are important in breaking bad news according to a recently conducted systematic literature review: emotional support; what and how much information to provide; manner of communicating news; and setting (43). The SPIKES protocol includes six steps for breaking bad news: (1) *setting* up the interview, (2) assessing the patient’s *perception*, (3) obtaining the patient’s *Invitation*, (4) giving *knowledge* and information to the patient, (5) addressing the patient’s *emotions* with empathic responses and (6) providing a s*trategy and summary of treatment options and future plans* (42). SPIKES was originally used in oncology but gradually adapted to other fields of medicine (42). Previous studies have identified the absence of specific guidelines for sharing difficult news in psychiatry (22) and showed the need for such protocols (23), yet the SPIKES model had not been empirically studied in FEP.

A in 2014 published qualitative study showed that psychiatrists in Israel experienced disclosure as problematic, unproductive and harmful (17). Factors as the uncertainty on the course and disclosure as a threat to a positive therapeutic alliance retain clinicians to give disclosure. This endorses the importance of working out a personalized diagnosis (in close consultation) with the individual and not focusing on the psychiatric label.

Participants views of what constitutes good communication during the diagnostic discussions were found to correspond to the stepwise framework of the SPIKES protocol (42): giving *Knowledge (step 4)* and information to the individuals, addressing the individuals *Emotions* (step 5) with empathic responses and *Strategy* and *Summary (step 6).* This last principle, *Strategy and Summary*, requires that the information is summarized and repeated and that the individuals is given an opportunity to voice any concerns or questions, especially given potential memory relapses. The clinicians and their patient should leave the conversation with a clear plan and time schedule for the next steps that need to be taken and the roles both will play in taking those steps. The SPIKES protocol could be a useful guide to initial diagnostic discussions; however, it probably needs some expansion/specifications to be appropriate for a mental health context, especially since communication of diagnostic information may be delivered over a number of support sessions, depending on the individual’s needs and memory capacities, and because stigma-related issues may need specific interventions, potentially also with loved ones, as a number of individuals indicated that their acceptance was important to their own acceptance. The reactions to diagnostic information vary between individuals. Therefore, an individualized personalized approach to provide diagnostic information is required.

Considering the current study results, a few limitations should be mentioned. This study was carried out with a small sample size of eleven men (73%) and four (27%) women. Although the sex distribution is not equal, it is comparable to the sex ratio in psychotic disorders (44). Cocchi et al. (44) have found that incident rates (cases per 100,000 per year) are consistently higher for males (median: 15.0) than for females (10.0), with a median rate ratio of 1.4. The fact that some participants (*n* = 6) had recently come out of the acute phase is informative. The qualitative design ensures that we collected rich experiences that, due to the limited respondent group, are not necessarily generalizable to the large group of people with first psychosis. Also, a qualitative design has a higher probability of researcher bias. We took this into account by having two researchers conducting the analysis, discussing the analysis in the whole research group, doing a member check and always backing up the results with quotes.

Thus, it is recommended to carry out additional studies involving more participants and encompassing different phases of a psychosis to gain detailed knowledge as to who needs what in which phase. Our study is retrospective and the primary disadvantage of this study design that it is possible that some respondents had difficulty remembering the first stage of treatment in which the diagnosis may have been discussed. It is indeed informative that they apparently remember little, which is an argument for repetition of the information after some time. This study is a two-center study in the north of the Netherlands and replication of this study in a multiple-center design with a comparable population is suggested. Nevertheless, this research has given new insights into the experiences and needs of individuals and research can be continued.

With these limitations in mind, this study has important implications for future research and for the development of guidelines in this area. Moran et al. (45) showed that psychiatrists found the disclosure of schizophrenia problematic, unproductive, and harmful (45). In our study we found that participants experienced the information as intense. Yet most participants still wanted the classification and withholding it was experienced as stigmatizing. It would be interesting to conduct a qualitative study with clinicians on experiences and barriers related to shared decision making to learn more about the reasons why we don’t ask our patients what they prefer regarding communicating diagnosis.

We also recommend EIS to develop guidelines to take the wishes and needs of individuals seriously and to really connect with the individual process of the individual with a first episode psychosis where information is provided at the right time, in the most appropriate way. These guidelines should cover the following aspects: *when* to provide information (addressing the moment of providing the patient’s diagnosis), *what* to provide (a providing a clarifying, tailor-made, descriptive diagnosis to formulate a personal narrative) and *how* to provide information to the patient and their loved ones (attitude and the way information is provided, e.g., providing the information in multiple ways: in a discussion and in writing). Furthermore, these guidelines should address how to pay attention to any emotional responses of the patient and/or their loved ones to the information.

## Data availability statement

The raw data supporting the conclusions of this article will be made available by the authors, without undue reservation.

## Ethics statement

The studies involving human participants were reviewed and approved by the University Medical Center Groningen. The patients/participants provided their written informed consent to participate in this study.

## Author contributions

NB, GP, and IH-O conducted the design of the study. MH conducted the research under supervision of NB, GP, and IH-O with assistance of BS and GR. NB and MH drafted the manuscript. NB, GP, IH-O, MH, BS, and GR constructed the design and revised the manuscript. All authors read and approved the final manuscript.
